# Immediate effects of smoking on optic nerve and macular perfusion measured by optical coherence tomography angiography

**DOI:** 10.1038/s41598-019-46746-z

**Published:** 2019-07-15

**Authors:** Maksym Ciesielski, Piotr Rakowicz, Marcin Stopa

**Affiliations:** Department of Ophthalmology, Chair of Ophthalmology and Optometry Heliodor Swiecicki University Hospital, Poznan University of Medical Sciences, 60-780 ul. Grunwaldzka 16/18, Poznan, Poland

**Keywords:** Outcomes research, Blood flow

## Abstract

The aim of this study was the assessment of the relationship between cigarette smoking and optic nerve and macular vessel density measured by optical coherence tomography angiography. We examined 30 eyes from 30 healthy habitual smokers. The examination was performed using a high-speed and high-resolution spectral-domain optical coherence tomography RTVue XR Avanti with AngioVue (Optovue, Fremont, CA, USA) with a split-spectrum amplitude-decorrelation angiography algorithm. Blood pressure, heart rate, vascular density in the area of the optic nerve head (4.5*4.5 mm) and vascular density with the foveal avascular zone in the central macula (3.0*3.0 mm) were measured and analyzed before, immediately after and 30 minutes after cigarette smoking. Quantitative measurements were carried out by AngioAnalytics Phase 7 software. Immediately after smoking both heart rate and blood pressure increased significantly (p < 0.001) compared to values before smoking and then significantly decreased after 30 minutes comparing to values obtained right after smoking (p < 0.001). The mean area of the foveal avascular zone, parafoveal vessel density, and peripapillary vessel density did not change significantly. Our results show no immediate influence of smoking on vessel density parameters measured by specific OCTA machine in healthy habitual smokers.

## Introduction

Imaging blood vessels in the posterior segment is essential in the diagnostics of retinal and choroidal vascular disorders. Since the introduction of the first methods of dye-based angiography in 1961, both technique and instrumentation have improved significantly^1^. The most recent method for visualization the fundus vessels of the eye is optical coherence tomography angiography (OCTA), an extension of the optical coherence tomography (OCT), which allows for non-invasive, non-contact, layered estimation of the structures of the eyeball. The first reports about OCT appeared in the 1990s from the Massachusetts Institute of Technology^2^. OCT has now become the basis for the diagnosis of the posterior segment of the eyeball and is a vital link in everyday clinical practice. OCTA compared to traditional angiography such as fluorescein angiography, and indocyanine angiography is non-invasive because no intravenous contrast is required^3,4^. Another advantage of this method is the possibility of analyzing superficial and deep retinal vasculature separately^5^. OCTA is useful in the diagnosis and treatment monitoring in several retinal and choroidal abnormalities^6,7^. These diseases include, but are not limited to, central serous chorioretinopathy^8^, age-related macular degeneration^9^, diabetic retinopathy^10^, and glaucoma^11^. Because of the high importance of OCTA in ophthalmic diagnosing, it is critical to perform it correctly with the exclusion of any possible factors that may affect its outcome. The effects of some of them, such as physical exercise^12^ or hyperoxia^13^ have been confirmed and described in the literature. The effects smoking has on the different organs’ blood flow vary significantly. It causes an acute increase in cerebral and coronary blood flow^14^, while in the skin and placenta, we can observe a blood flow decrease^15^. This paper analyzes the immediate effect of smoking on the optic nerve and macular perfusion measured by specific OCTA machine in healthy habitual smokers. Although the effect of smoking on the vascular network of optic disc and macula has already been investigated using various methods, including fluorescein angiography^16–18^ and OCTA, we believe that by considering additional factors, our study may result in a more accurate description of the influence of nicotine on the fundus circulation.

## Materials and Methods

### Study population

Thirty healthy volunteers (30 eyes) with no history of any systemic or ocular diseases except for refractive error participated in this prospective study. Before inclusion in the study group, we performed a preliminary ophthalmic examination. We excluded those, who had increased intraocular pressure (>21 mmHg in Goldmann applanation tonometry) and visual acuity less than 20/20 (best corrected visual acuity measurement with Early Treatment Diabetic Retinopathy Study chart). Additionally, we have divided the research group into those who have been smoking for less than 10 years (14 participants) and for those who have been smoking for over 10 years (16 participants). The research was conducted according to the Declaration of Helsinki and was approved by the medical ethics committee of Poznan University of Medical Sciences. Each patient had to sign an informed consent after the nature of the study had been fully explained.

### Examination

Each patient was asked to abstain from smoking for at least one hour before the examination. Moreover, we asked each participant not to take any physical activity for 15 minutes before starting measurements. All volunteers were asked to smoke up the whole amount of one cigarette, and all cigarettes were of the same brand in order to standardize the nicotine content (0.6 mg per one cigarette). Three series of examinations were carried out, including systemic blood pressure and heart rate measurements and OCTA-scans, one before and the others instantly and 30 minutes after smoking. We used the following equation to determine the mean blood pressure (MBP):

$${\rm{Mean}}\,{\rm{blood}}\,{\rm{pressure}}=1/3({\rm{systolic}}\,{\rm{blood}}\,{\rm{pressure}}-{\rm{diastolic}}\,{\rm{blood}}\,{\rm{pressure}})+{\rm{diastolic}}\,{\rm{blood}}\,{\rm{pressure}}.$$

### OCT angiography

All eyes in our study were imaged using RTVue XR Avanti with AngioVue Comprehensive imaging software, which is based on split-spectrum amplitude-decorrelation angiography (SSADA) algorithm. This algorithm was created to reduce scanning time. It detects motion in the blood vessel lumen by measuring the variation in reflected OCT signal amplitude between consecutive cross-sectional scans^19^. All imaging were obtained sequentially at a standardized time between 3 PM and 6 PM to reduce the potential influence of diurnal variation of some ocular parameters, especially choroidal thickness^20^. Each participant had examined one eye only, which was randomly selected.

The macula was imaged three times using a 3 mm × 3 mm scan, and the optic disc region was also imaged three times using a 4,5 mm × 4,5 mm scan. Each OCT angiography volume contained 304 · 304 A-scans with two consecutive B-scans that were captured at each fixed position, with two orthogonal OCTA volume scans (horizontal and vertical) acquired to minimize motion artifacts. Within the macula, both foveal avascular zone (FAZ) and macular vessel density were scanned. The optic nerve disc was evaluated for vascular density. The whole procedure was repeated in the same manner instantly and 30 minutes after smoking. The retinal vasculature was segmented into two layers by an automated retinal layer segmentation algorithm installed on the OCTA device. The superficial vascular plexus included vessels running in the nerve fiber layer (NFL), the ganglion cell layer (GCL) and the inner plexiform layer (IPL), while the deep plexus included vessels in the inner nuclear layer (INL) and the outer plexiform layer (OPL). During vascular density analysis, the software analyzed both vascular layers overlapping on each other. Quantitative analysis was performed on the OCTA using the AngioAnalytics Phase 7 software. Vascular density was presented as a percentage of the particular area occupied by vessels in the en face projection and was automatically calculated by the software.

One of the limitations of OCTA is a proclivity for image artifacts due to patient movement or blinking. Scans with motion artifacts contain lines or gaps that make it impossible to evaluate images accurately^21^. In view of the above, only images with no motion artifacts and signal strength index >60 were included in our analysis.

### Statistical analysis

All measurements were gathered and managed using Microsoft Excel 2017. PSPP 0.10.4 MS Office 2013 was used to perform the statistical analysis. The mean of the three measurements before and after cigarette smoking was compared using t-tests for paired data. To evaluate the degree of correlation between two variables we used Pearson correlation coefficient. The significant level was set to 0.05.

### Ethical approval and informed consent

All procedures performed in studies involving human participants were in accordance with the ethical standards of the institutional research committee and with the 1964 Helsinki declaration and its later amendments or comparable ethical standards. Informed consent was obtained from all individual participants included in the study.

## Results

The demographic charecteristics of the study population are shown in Table 1. Ages of the subjects ranged from 23 to 43 years (mean ± SD, 32.53 ± 5.87). The number of cigarettes smoked per day varied between 8 and 25 (mean ± SD, 14 ± 4). The patients’ history of smoking ranged from 2 to 30 years (mean ± SD, 12.03 ± 6.9). The women to men ratio was 12:18.

**Table 1:** Characteristics of the study population. (n = 30).

Mean age (years ± SD)	32.53 ± 5.87
Gender (female:male)	12:18
Length of smoking (years ± SD)	12 ± 6.9
Number of cigarettes smoked per day (n ± SD)	14 ± 4

All the cardiovascular parameters (SBP, DBP, MBP, HR) increased significantly after smoking. We also noticed a significant decrease in these parameters 30 minutes later according to the measurements carried out instantly after smoking. Detailed results are given in Table 2.

**Table 2:** Cardiovascular parameters before, instantly after and 30 minutes after smoking.

	Mean ± SD (P)		
	Before smoking	Immediately after	After 30 min
Systolic blood pressure (mmHg)	129.8 ± 11.5	147.5 ± 10.4 (<0.001)	132.9 ± 12.1 (<0.001)
Diastolic blood pressure (mmHg)	79.2 ± 9.4	85 ± 9.1 (<0.001)	78.4 ± 8.1 (<0.001)
Mean blood pressure (mmHg)	96.1 ± 9.1	105.6 ± 8.0 (<0.001)	96.4 ± 8.4 (<0.001)
Heart rate (1/min)	74.9 ± 10.7	92.6 ± 13.9 (<0.001)	77.6 ± 11.3 (<0.001)

**Figure 1:**
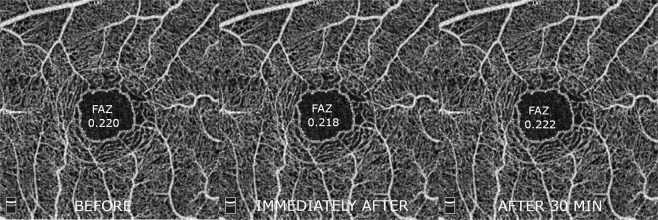
A representative example of 3 mm × 3 mm foveal OCTA scans showing the area of measurement with foveal avascular zone values obtained before, immediately after and 30 minutes after smoking.

**Figure 2:**
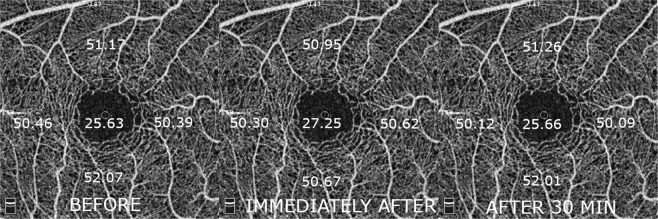
A representative example of 3 mm × 3 mm foveal OCTA scans showing the area of measurement with vessel density values for each sector obtained before, immediately after and 30 minutes after smoking.

The average size of the foveal avascular zone (FAZ) did not change significantly. There were also no significant changes in vascular density measured for the macular region (Table 3). Both FAZ and macular vascular density values are also shown in Figs 1 and 2, respectively.

**Table 3:** FAZ and macular flow density before, instantly after and 30 min after smoking.

	Mean ± SD (P)		
	Before smoking	Immediately after	After 30 minutes
FAZ (mm^2^)	0.220 ± 0.1	0.218 ± 0.1 (0,917)	0.222 ± 0.1 (0.866)
**Macular flow density (%):**			
Whole en face	49.35 ± 2.1	49.01 ± 2.1 (0.529)	48.88 ± 2.3 (0.818)
Fovea	25.63 ± 6.7	27.25 ± 6.6 (0.349)	25.66 ± 6.3 (0.343)
Parafovea	51.01 ± 2.1	50.75 ± 2.2 (0.644)	50.75 ± 3.1 (0.995)
Temporal	50.39 ± 2.4	50.62 ± 2.1 (0.695)	50.09 ± 3.1 (0.445)
Superior	51.17 ± 2.9	50.95 ± 2.5 (0.754)	51.26 ± 3.4 (0.688)
Nasal	50.46 ± 2.6	50.30 ± 3.5 (0.845)	50.12 ± 4.0 (0.852)
Inferior	52.07 ± 2.5	50.67 ± 3.2 (0.620)	52.01 ± 3.5 (0.721)

**Figure 3:**
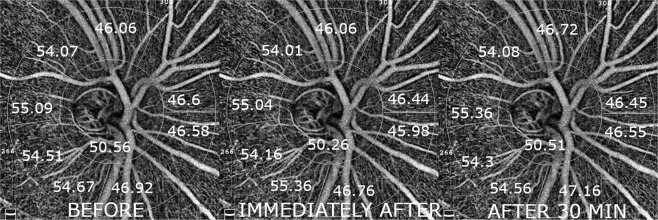
A representative example of 4.5 mm × 4.5 mm peripapillary OCTA scans showing the area of measurements with vessel density values for each sector obtained before, immediately after and 30 minutes after smoking.

Vascular density measurements of the optic disc region showed no significant changes (Table 4, Fig. 3). There was also no statistically significant correlation between cardiovascular parameters changes and any measured vascular parameters.

**Table 4:** Peripapillary flow density before, instantly after and 30 min after smoking.

	Mean % ± SD (P)		
	Before smoking	Immediately after	After 30 minutes
Whole en face	47.83 ± 3.6	47.61 ± 3.8 (0.818)	47.85 ± 3.3 (0.798)
Inside disc	50.56 ± 4.2	50.26 ± 3.7 (0.771)	50.51 ± 4,2 (0.809)
Peripapillary	49.5 ± 4.5	49.26 ± 4.6 (0.836)	49.63 ± 4.4 (0.754)
Peripapillary nasal superior	46.6 ± 6.4	46.44 ± 6.9 (0.923)	46.45 ± 6.3 (0.993)
Peripapillary nasal inferior	46.58 ± 7.0	45.98 ± 6.7 (0.734)	46.55 ± 6.7 (0.740)
Peripapillary inferior nasal	46.92 ± 6.2	46.76 ± 5.8 (0.921)	47.16 ± 6.4 (0.804)
Peripapillary inferior temporal	54.67 ± 5.2	55.36 ± 4.9 (0.596)	54.56 ± 4.1 (0.499)
Peripapillary temporal inferior	54.51 ± 3.4	54.16 ± 4.2 (0.725)	54.3 ± 3.6 (0.891)
Peripapillary temporal superior	55.09 ± 3.1	55.04 ± 3.7 (0.955)	55.36 ± 3.6 (0.737)
Peripapillary superior temporal	54.07 ± 4.3	54.01 ± 3.9 (0.959)	54.08 ± 4.3 (0.947)
Peripapillary superior nasal	46.06 ± 6.2	46.06 ± 6.1 (0.998)	46.72 ± 6.2 (0.678)

Additionally, there was no difference before and after smoking in macular and optic nerve disc vascular parameters in individuals who have been smoking for less than 10 years (47% of patients) and more than 10 years (53% of patients). Detailed results are presented in Tables 5 and 6.

**Table 5:** FAZ, macular and peripapillary vessel density before, instantly after and 30 min after smoking (smoking length < 10 years).

	Mean ± SD (P)		
	Before smoking	Immediately after	After 30 minutes
FAZ (mm^2^)	0.221 ± 0.1	0.217 ± 0.1 (0.9)	0.220 ± 0.1 (0.92)
**Macular flow density (%):**			
Whole en face	49.03 ± 2.2	48.42 ± 2.2 (0.472)	48.71 ± 1.6 (0.696)
Fovea	26.49 ± 7.3	27.88 ± 7.6 (0.623)	26.46 ± 6.8 (0.608)
Parafovea	51.00 ± 2.0	50.69 ± 2.4 (0.713)	50.5 ± 2.2 (0.825)
Temporal	50.29 ± 3.1	50.41 ± 2.5 (0.915)	49.86 ± 2.4 (0.562)
Superior	51.14 ± 2.0	51.14 ± 2.9 (0.994)	50.86 ± 2.6 (0.786)
Nasal	50.61 ± 2.5	50.93 ± 2.9 (0.759)	50.26 ± 3.4 (0.580)
Inferior	52.02 ± 2.6	51.24 ± 3.4 (0.499)	51.40 ± 3.7 (0.903)
**Peripapillary flow density (%):**			
Whole en face	47.57 ± 4.4	47.51 ± 4.3 (0.969)	47.54 ± 3.6 (0.981)
Inside disc	50.86 ± 4.5	50.66 ± 4.0 (0.901)	50.53 ± 4.9 (0.939)
Peripapillary	49.16 ± 5.4	49.44 ± 5.3 (0.891)	49.38 ± 4.7 (0.973)
Peripapillary nasal superior	46.36 ± 7.5	46.76 ± 7.5 (0.890)	46.01 ± 6.7 (0.784)
Peripapillary nasal inferior	46.43 ± 7.8	46.3 ± 6.8 (0.963)	46.44 ± 6.4 (0.957)
Peripapillary inferior nasal	47.14 ± 5.7	48.32 ± 4.1 (0.535)	48.24 ± 4.8 (0.963)
Peripapillary inferior temporal	55.44 ± 3.9	57.31 ± 3.9 (0.214)	54.84 ± 3.0 (0.072)
Peripapillary temporal inferior	56.16 ± 2.8	55.99 ± 4.0 (0.897)	56.03 ± 3.0 (0.975)
Peripapillary temporal superior	55.46 ± 3.3	55.87 ± 3.7 (0.757)	55.76 ± 3.6 (0.938)
Peripapillary superior temporal	53.85 ± 4.7	54.26 ± 4.2 (0.811)	53.73 ± 4.1 (0.740)
Peripapillary superior nasal	46.96 ± 5.7	48.16 ± 5.0 (0.559)	48.08 ± 4.8 (0.963)

**Table 6:** FAZ, macular and peripapillary vessel density before, instantly after and 30 min after smoking (smoking length > 10 years).

	Mean ± SD (P)		
	Before smoking	Immediately after	After 30 minutes
FAZ (mm^2^)	0.222 ± 0.1	0.220 ± 0.1 (0.964)	0.222 ± 0.1 (0.959)
**Macular flow density (%):**			
Whole en face	49.63 ± 1.9	49.53 ± 1.9 (0.883)	49.03 ± 2.8 (0.564)
Fovea	24.89 ± 6.3	25.89 ± 5.8 (0.642)	24.96 ± 5.9 (0.656)
Parafovea	51.01 ± 2.3	50.79 ± 2.2 (0.783)	50.97 ± 3.7 (0.872)
Temporal	50.47 ± 1.7	50.8 ± 1.7 (0.592)	50.3 ± 3.6 (0.623)
Superior	51.19 ± 3.6	50.78 ± 2.2 (0.694)	51.61 ± 4.0 (0.473)
Nasal	50.33 ± 2.7	49.75 ± 4.0 (0.637)	50.0 ± 4.5 (0.869)
Inferior	52.11 ± 2.5	52.09 ± 3.1 (0.990)	52.54 ± 3.4 (0.705)
**Peripapillary flow density (%):**			
Whole en face	48.06 ± 2.9	47.7 ± 3.4 (0.754)	48.11 ± 3.2 (0.726)
Inside disc	50.29 ± 4.1	49.91 ± 3.6 (0.778)	50.49 ± 3.8 (0.657)
Peripapillary	49.8 ± 3.7	49.1 ± 4.0 (0.612)	49.84 ± 4.3 (0.616)
Peripapillary nasal superior	46.81 ± 5.6	46.16 ± 6.7 (0.765)	46.83 ± 6.0 (0.766)
Peripapillary nasal inferior	46.71 ± 6.4	45.7 ± 6.8 (0.669)	46.66 ± 7.1 (0.699)
Peripapillary inferior nasal	46.73 ± 6.8	45.4 ± 6.8 (0.584)	46.21 ± 7.6 (0.754)
Peripapillary inferior temporal	53.99 ± 6.1	53.66 ± 5.2 (0.870)	54.32 ± 5.0 (0.715)
Peripapillary temporal inferior	53.08 ± 3.3	52.57 ± 3.8 (0.691)	52.79 ± 3.5 (0.863)
Peripapillary temporal superior	54.76 ± 3.0	54.31 ± 3.7 (0.703)	55.0 ± 3.7 (0.602)
Peripapillary superior temporal	54.26 ± 4.0	53.8 ± 3.7 (0.741)	54.39 ± 4.6 (0.688)
Peripapillary superior nasal	45.26 ± 6.6	44.22 ± 6.6 (0.657)	45.54 ± 7.2 (0.592)

## Discussion

Our results are one of few which show the lack of immediate effects of cigarette smoking on the vascular density of central retina and optic disc region in healthy habitual smokers obtained by OCTA. However, it should be noted that we used one, specified OCT machine and scanned only two small fragments of retinal vasculature (3.0*3.0 mm of the central macula and 4.5*4.5 mm peripapillary). Despite significant changes in the parameters determining the work of the cardiovascular system, we did not observe parallel changes in parameters reflecting the retinal circulation.

The human retina draws oxygen and nutrients from two separate blood systems. Choriocapillaris are the first of them and supplies the outer half of the retina. The inner half of the retina is vascularized through the branches of the central retinal artery, and we focused only on this part of retinal vascularization in our study. Its larger vessels lay most superficially, in the nerve fiber layer close to the inner limiting membrane. These vessels prolongate and divide eventually into two-layered plexus of capillaries relating to the current OCT nomenclature^22^. The superficial layer is located in the nerve fiber layer, ganglion cell layer, and inner plexiform layer, while the deeper one runs in the inner nuclear layer and outer plexiform layer. Histologically, arterial walls within the retina have a different structure than arteries of the same size in other tissues mainly because of a more developed layer of smooth muscles^23,24^.

The capillary wall is composed of three distinct elements: endothelial cells, intramural pericytes, and a basement lamina. Along with the optic nerve head and within the retina, α- and β-adrenergic receptors and receptors for angiotensin II are present^25^. These receptors are involved in the formation of vascular changes under the influence of various factors, such as the increase in blood pressure.

The autoregulation of circulation in the human body is complex, and many factors make it possible to maintain constant blood flow despite a variable perfusion pressure. The process of autoregulation depends, among other things, on the local miogenic response, endothelium-derived substances, local metabolic factors and the autonomic nerves^26^. It is still unclear what are the precise mechanisms underlying retinal blood flow autoregulation, but we can assume that a balanced contribution between myogenic and metabolic factors is essential for its proper functioning.

It has long been known that while smoking cigarettes, the level of norepinephrine and epinephrine in the blood increases significantly^27^. This leads to an increase in blood pressure, which should result in autoregulation process. It turns out that the autoregulation process starts only after reaching a specific value of the arterial pressure. In one study there was no detectable change in retinal blood flow until the mean brachial artery blood pressure was elevated to an average of 115 mm Hg, which represented an average rise in mean blood pressure of 41% above baseline values^28^. In our opinion, this could explain the fact that we observed no changes in flow density in our patients.

By analyzing the various studies described in the literature, inconsistent results can be noticed. In 1985 Robinson *et al*. estimated the acute effect of cigarette smoking by using the blue field simulation technique and showed an increased macular capillary blood flow expressed by increased leukocyte velocity suggesting a significant increase in vascular resistance in examined tissues^16^. It is not possible to compare these results directly with our data since flow velocity obtained with the blue field simulation technique and vessel density measured by OCTA are dissimilar parameters. However, we can assume that if the vascular resistance increases, the same results obtained by OCTA should be the decrease in the vascular density resulting from the vessels contraction. Our data do not prove that assumption. In the research from 1985, the percentage increase of mean arterial blood pressure was 6,57% contrary to 10,1% in our results. Additionally, it is noteworthy that nicotine content (0.13–1.22 mg) in Robinsons’ study from 1985 differed notably from cigarettes in our experiments (0.6 mg) and the number of the research group was more than two times smaller than ours.

The short-term effect of blood pressure increase caused by a series of physical exercises on blood flow in the macula and the optic disc measured by OCTA was described in the literature^12^. They showed decreased blood vessels density both in macular and optic disc region. We expected similar results. The surprising observation was that no change in peripapillary vessel density occurred after smoking and elevation of blood pressure and heart rate. The explanation for that could be similar to the previous example discussed above. The increase of systemic circulation parameters caused by physical exercise was almost three times higher (SBP – 31%, HR – 60%) than in the case of cigarette smoking (SBP – 10,1%, HR – 24%). Probably, the degree of blood pressure increase due to physical exercises was enough to initiate an autoregulation process, which led in this case to constriction of blood vessels. In our study, the increase in blood pressure was probably too small to initiate the autoregulation process, which translated into no changes in flow density in OCTA scans.

Recently, two articles have been published that raise the issue of the influence of smoking on retinal vascular parameters measured with OCTA. The results obtained by Ayhan *et al*.^29^ showed that smoking the cigarette does not cause significant changes in the size of the FAZ area and the macular vessel density in healthy habitual smokers. The authors did not evaluate the peripapillary vessel density. Holló G. suggests that both macular and peripapillary vessel density values in healthy middle-aged smokers are not influenced by acute smoking a cigarette^30^, but the study group was four times smaller than ours and subjects were asked to smoke a cigarette of the brand they usually smoke so the nicotine content has not been standardized. Our results are in line with these above. However, it is worth emphasizing that only in our study the systemic circulation parameters such as arterial pressure and heart rate were parallel analyzed.

It should be noted that some limitations may apply. First of all, we admit that each smoker might have their unique manner of smoking, but no restrictions about this have been suggested besides the cigarette brand. The effects of nicotine last from 5 min to 2 hours, but during the first 2 min of nicotine absorption, the arterial concentrations range 6–10 folds^31^. To avoid possible differences in nicotine levels, we asked all participants to smoke up the cigarette of the same brand in the smoking area next to the research room. Thanks to this the time from finishing smoking to starting the examination was less than 2 minutes so we could examine patients at the time when the concentration of nicotine in blood should be the highest. Moreover, we did not know if one hour of refraining from smoking before the test was enough to exclude the effect of smoking on systemic and retinal blood flow because no research was available. However, our preliminary results indicated that the values obtained 30 min after smoking had compared to values obtained before smoking. The above was observed in participants who abstained from smoking either for one hour and for more than one hour irrespectively. Therefore, we could assume, that one hour after smoking was enough to eliminate the effect of nicotine on systemic and retinal blood flow. However, each habitual smoker has their own daily smoking cycle and there is always a zone of nicotine concentrations present in this cycle in which the smoker is comfortable and that a cigarette consumption produces a short term spike in the above concentration^32^. In addition, we must take into account that the nicotine content of our cigarettes was relatively low. Smoking cigarettes or cigars with unusually high nicotine levels can indeed extend the time required for cardiovascular parameters to stabilize.

Another problem is that in our group we recruited only habitual smokers so when assessing the immediate effects of smoking on vessel density, we should hypothesize, that there are differences in nicotine tolerance between smokers and non-smokers. A solution to the shortcoming would be to enroll age and sex-matched nonsmoker subjects as control, but encouraging healthy, non-smoking participants to smoke would raise controversy over ethical background and recruitment of such control group could be very difficult.

It is worth noting that the process of autoregulation is complex. Its analyzing might be insufficient based only on the data obtained thanks to OCTA because we cannot evaluate the blood flow velocity. Omae *et al*. claim that after smoking the retinal blood flow and the blood velocity significantly decrease without any changes in vessels’ diameter, but they used other methods to assess retinal circulation, which may be more sensitive to detect changes caused by smoking than OCTA^33^. This circumstance could be the reason why we did not observe any significant changes in vessel density on OCTA scans.

We realize that there are other factors like ocular perfusion pressure that might play a role when assessing changes in retinal vessel density. We did not measure that parameter. However, we agree that it would provide valuable data in this context and feel that it could and should be further explored in future studies.

Furthermore, we realize that much larger sample size would be necessary to give results with greater precision and power since the observed effects of smoking are small. In addition, the differences between patients age, duration of smoking and the number of cigarettes smoked per day may affect the different course of autoregulation due to possible vascular changes in those, who are older and has been smoking more and longer. Furthermore we examined healthy individuals in our study. It is also not clear if the presence of some coexisting ocular or systemic conditions might trigger changes in macular and peripapillary vessels density in OCTA. These topics are deferred to future work.

Our study shows no immediate effects of single-cigarette smoking in healthy habitual smokers on foveal avascular zone, macular vessel density in the central 3*3 mm area and peripapillary vessel density in the area of 4.5*4.5 mm obtained by specific OCTA machine. It also proves that changes in systemic circulation parameters caused by single-cigarette smoking in healthy habitual smokers are not reflected in changes in vascular parameters obtained in OCTA.
